# Supplementary material for: The mental health prognosis of offspring born of genocidal rape is influenced by family members, the community and their perceptions toward them
Source: PLoS One. 2024 Apr 30;19(4):e0302330. doi: 10.1371/journal.pone.0302330 (PMC11060550; doi:10.1371/journal.pone.0302330)
Supplement: S2 File — (PDF) [file pone.0302330.s002.pdf]

Human Participants Research Checklist

Complete the following if your study involved human participants or human participants' data. These questions should be addressed for prospective and retrospective studies.

1. Did you obtain ethics approval for this study?

   - If yes, please upload (file type "Other") the original approval document you received from your ethics committee. If the original document is in another language, please also provide an English translation.

     _X__ Uploaded ___ N/A

   - If you did not obtain ethical approval, please explain why this was not required below.

     NA

2. If you prospectively recruited human participants for the study – for example, you conducted a clinical trial, distributed questionnaires, or obtained tissues, data or samples for the purposes of this study, please report in the Methods:

   i. the day, month and year of the **start and end** of the recruitment period for this study.

   ii. whether participants provided informed consent, and if so, what type was obtained (for instance, written or verbal, and if verbal, how it was documented and witnessed). If your study included minors, state whether you obtained consent from parents or guardians. If the need for consent was waived by the ethics committee, please include this information.

   ___ Completed **_X_ N/A**

3. If you are reporting a retrospective study of medical records or archived samples, please report in the Methods section:

   i. the day, month and year when the data were accessed for research purposes

   ii. whether authors had access to information that could identify individual participants during or after data collection

   ___ Completed **_X_ N/A**

UNIVERSITY of RWANDA

COLLEGE OF MEDICINE AND HEALTH SCIENCES

DIRECTORATE OF RESEARCH & INNOVATION

CMHS INSTITUTIONAL REVIEW BOARD (IRB)

Kigali, the 4th/June/2021

NYIRANDAMUTSA Fortunée

School of Medicine and Pharmacy, UR

Notice of Renewal of Approval for Research Project: No 174 /CMHS IRB/2021

Your Project title ***"Early Childhood Trauma and Mental Health Prognosis: The impact of Rape Trauma on Parenting"*** has been evaluated by CMHS Institutional Review Board.

		Involved in the decision		
			No (Reason)	
**Name of Members**	**Institute**	**Yes**	**Absent**	**Withdrawn from the proceeding**
Prof Kato J. Njunwa	UR-CMHS	X		
Dr Stefan JANSEN	UR-CMHS	X		
Dr Brenda Asiimwe-Kateera	UR-CMHS	X		
Prof Ntaganira Joseph	UR-CMHS	X		
Dr Tumusiime K. David	UR-CMHS	X		
Dr Kayonga N. Egide	UR-CMHS	X		
Mr Kanyoni Maurice	UR-CMHS		X	
Prof Munyanshongore Cyprien	UR-CMHS	X		
Mrs Ruzindana Landrine	Kicukiro district		X	
Dr Gishoma Darius	UR-CMHS	X		
Dr Donatilla Mukamana	UR-CMHS	X		
Prof Kyamanywa Patrick	UR-CMHS		X	
Prof Condo Umutesi Jeannine	UR-CMHS		X	
Dr Nyirazinyoye Laetitia	UR-CMHS	X		
Dr Nkeramihigo Emmanuel	UR-CMHS		X	
Sr Maliboli Marie Josee	CHUK	X		
Dr Mudenge Charles	Centre Psycho-Social	X		

After reviewing your protocol, **Continuation of Approval has been granted to your study.**

Please note that approval of the protocol and consent form is valid for **12 months**.

UNIVERSITY of RWANDA

COLLEGE OF MEDICINE AND HEALTH SCIENCES

DIRECTORATE OF RESEARCH & INNOVATION

CMHS INSTITUTIONAL REVIEW BOARD (IRB)

Kigali, the 4th/June/2021

NYIRANDAMUTSA Fortunée

School of Medicine and Pharmacy, UR

Notice of Renewal of Approval for Research Project: No 174 /CMHS IRB/2021

Your Project title ***"Early Childhood Trauma and Mental Health Prognosis: The impact of Rape Trauma on Parenting"*** has been evaluated by CMHS Institutional Review Board.

		Involved in the decision		
			No (Reason)	
**Name of Members**	**Institute**	**Yes**	**Absent**	**Withdrawn from the proceeding**
Prof Kato J. Njunwa	UR-CMHS	X		
Dr Stefan JANSEN	UR-CMHS	X		
Dr Brenda Asiimwe-Kateera	UR-CMHS	X		
Prof Ntaganira Joseph	UR-CMHS	X		
Dr Tumusiime K. David	UR-CMHS	X		
Dr Kayonga N. Egide	UR-CMHS	X		
Mr Kanyoni Maurice	UR-CMHS		X	
Prof Munyanshongore Cyprien	UR-CMHS	X		
Mrs Ruzindana Landrine	Kicukiro district		X	
Dr Gishoma Darius	UR-CMHS	X		
Dr Donatilla Mukamana	UR-CMHS	X		
Prof Kyamanywa Patrick	UR-CMHS		X	
Prof Condo Umutesi Jeannine	UR-CMHS		X	
Dr Nyirazinyoye Laetitia	UR-CMHS	X		
Dr Nkeramihigo Emmanuel	UR-CMHS		X	
Sr Maliboli Marie Josee	CHUK	X		
Dr Mudenge Charles	Centre Psycho-Social	X		

After reviewing your protocol, **Continuation of Approval has been granted to your study.**

Please note that approval of the protocol and consent form is valid for **12 months**.

Email: researchcenter@ur.ac.rw P.O Box 3286 Kigali, Rwanda www.ur.ac.rw

You are responsible for fulfilling the following requirements:

1. Changes, amendments, and addenda to the protocol or consent form must be submitted to the committee for review and approval, prior to activation of the changes.
2. Only approved consent forms are to be used in the enrollment of participants
3. All consent forms signed by subjects should be retained on file. The IRB may conduct audits of all study records, and consent documentation may be part of such audits.
4. A continuing review application must be submitted to the IRB in a timely fashion and before expiry of this approval.
5. Failure to submit a continuing review application will result in termination of the study.
6. Notify the Rwanda National Ethics committee once the study is finished.

Sincerely,

Date of Approval: June 4th, 2021
Expiration date: June 4th, 2022

Dr Stefan JANSEN
Ag Chairperson Institutional Review Board,
College of Medicine and Health Sciences, UR

Cc:

- Principal College of Medicine and Health Sciences, UR
- University Director of Research and Innovations, UR

Email: researchcenter@ur.ac.rw P.O Box 3286 Kigali, Rwanda www.ur.ac.rw

You are responsible for fulfilling the following requirements:

1. Changes, amendments, and addenda to the protocol or consent form must be submitted to the committee for review and approval, prior to activation of the changes.
2. Only approved consent forms are to be used in the enrollment of participants
3. All consent forms signed by subjects should be retained on file. The IRB may conduct audits of all study records, and consent documentation may be part of such audits.
4. A continuing review application must be submitted to the IRB in a timely fashion and before expiry of this approval.
5. Failure to submit a continuing review application will result in termination of the study.
6. Notify the Rwanda National Ethics committee once the study is finished.

Sincerely,

Date of Approval: June 4th, 2021
Expiration date: June 4th, 2022

Dr Stefan JANSEN
Ag Chairperson Institutional Review Board,
College of Medicine and Health Sciences, UR

Cc:

- Principal College of Medicine and Health Sciences, UR
- University Director of Research and Innovations, UR
