# Supplementary material for: The mental health prognosis of offspring born of genocidal rape is influenced by family members, the community and their perceptions toward them
Source: PLoS One. 2024 Apr 30;19(4):e0302330. doi: 10.1371/journal.pone.0302330 (PMC11060550; doi:10.1371/journal.pone.0302330)
Supplement: Attachment — Submitted filename: Response to Reviewers.doc [file pone.0302330.s003.doc]

**Rebuttal letter**

We wish to express our sincere appreciation to the editor and reviewer for their insightful comments. We have done our best to address all the issues raised and we hope that the final version of this manuscript is much improved due to their comments.

**Note:** This table corresponds to the manuscript with track changes. It means that page numbers correspond to the manuscript with track changes.

| **N** | **Reviewer 1** | **Reviewer’s comments** | **Page and line on which addressed** | **How comments were addressed** |
| --- | --- | --- | --- | --- |
|  | **General comments** | **Comments to the Author**  1. Is the manuscript technically sound, and do the data support the conclusions? The manuscript must describe a technically sound piece of scientific research with data that supports the conclusions. Experiments must have been conducted rigorously, with appropriate controls, replication, and sample sizes. The conclusions must be drawn appropriately based on the data presented.  Reviewer #1: Yes  Reviewer #2: Yes  2. Has the statistical analysis been performed appropriately and rigorously?  Reviewer #1: Yes  Reviewer #2: Yes  3. Have the authors made all data underlying the findings in their manuscript fully available?  The [PLOS Data policy](http://www.plosone.org/static/policies.action#sharing) requires authors to make all data underlying the findings described in their manuscript fully available without restriction, with rare exception (please refer to the Data Availability Statement in the manuscript PDF file). The data should be provided as part of the manuscript or its supporting information, or deposited to a public repository. For example, in addition to summary statistics, the data points behind means, medians and variance measures should be available. If there are restrictions on publicly sharing data—e.g. participant privacy or use of data from a third party—those must be specified.  Reviewer #1: Yes  Reviewer #2: No  4. Is the manuscript presented in an intelligible fashion and written in standard English?  PLOS ONE does not copyedit accepted manuscripts, so the language in submitted articles must be clear, correct, and unambiguous. Any typographical or grammatical errors should be corrected at revision, so please note any specific errors here.  Reviewer #1: Yes  Reviewer #2: Yes | Whole document | We really appreciate the reviewer 1 and reviewer 2 for the overall appreciation of the quality of this manuscript.  We have made the data fully available. The following statement was added in the manuscript:  All relevant data are within the manuscript and its supporting information files (via the link: <https://zenodo.org/records/10495470>). |
| 1 | Reviewer #1: | Reviewer #1: Overall the research paper impressively delves into a crucial topic but I would like to share few recommendations. 1. Paper should include a more robust theoretical background. Strengthening this aspect would provide a deeper foundation for the study contributing to a more comprehensive understanding of the study variables. | Page 2,  line 8 | **We appreciate the reviewer for the constructive comments, we have included four different theories that may help to further explanation of the factors in our introduction as follows:**  ***Introduction***  *Rape was a major issue during the 1994 genocide against the Tutsi, and its results had long-term severe effects on the mental health and psychosocial living conditions of the victims and the offspring born of this sexual violence [1]. During the Rwandan genocide, men, predominantly Hutu, utilized the rape of women, primarily Tutsi, as a political weapon to exterminate the Tutsi ethnic group [2]. Drawing upon trauma theory[3], which illuminates the profound impact of traumatic events, we understand the enduring psychological wounds inflicted during this period. Originating from the works of psychoanalyst Sigmund Freud, trauma theory posits that exposure to severe, distressing events can have lasting psychological impacts on individuals. In the context of genocidal rape, trauma theory helps us understand the deep and lasting effects on the mental health of both the victims and their offspring, emphasizing the interconnectedness of individual and collective trauma.*  *Between April 6 and July 12, 1994, Tutsi girls and women endured widespread rape, particularly intensifying after mid-May when Hutu officials directed the Interahamwe ("those who stand together") militia not to spare Tutsi women and children in the genocide [2]. This period witnessed the utilization of genocidal rape to humiliate, kill and eradicate the Tutsi as an ethnic group [4,5]. Social cognitive theory, developed by Albert Bandura, provides a lens to analyse how family and community attitudes shape the self-perception of children born of genocidal rape, exploring the observational learning and socialization processes influencing their coping strategies. In the context of children born of genocidal rape, this theory helps us understand how their self-perception is influenced by the attitudes and behaviours they observe in their family and community.*  *Though the exact number of children born as a result of genocide rape remains unknown, it is estimated that between 2,000 and 10,000 offspring [6,7], were born from 250,000 to 500,000 estimated rapes of Tutsi women and girls [7,8]. Many of these children, born of multiple and gang rapes, find themselves disconnected from their fathers and the families from which they came [9]. The secrecy surrounding their origins, rooted in the mothers' difficulty in disclosing the circumstances of their children's birth, poses challenges [9]. Using ecological systems theory[10], developed by Urie Bronfenbrenner, we examine the multifaceted influences on mental health at individual, family, community, and societal levels. This theory posits that individuals are influenced by multiple interconnected systems, including the microsystem (individual relationships), mesosystem (interactions between microsystems), exosystem (external influences on microsystems), macrosystem (cultural values), and chronosystem (temporal changes). Applied to the context of children born of genocidal rape, this theory allows us to explore how various environmental factors at different levels impact their mental health and coping mechanisms.*  *[9]The offspring born of genocidal rape face profound challenges, including societal violent abuse resulting from their birth and the lack of stable familial networks [11]. Health issues and developmental challenges further compound their struggles, with mothers' psycho-social trauma influencing early childhood and adulthood development [11]. Stigmatization[12,13] and identity[14] theories, developed by Erving Goffman and Erik Erikson, respectively, shed light on how societal labels and perceptions impact the self-identity and well-being of these children. Stigmatization theory explores how individuals with socially stigmatized attributes are treated by society. Applied to the children born of genocidal rape, this theory helps us understand the societal biases and abuse they face due to being labelled as the perpetrator's offspring. Identity theory, on the other hand, emphasizes the stages of identity development individuals go through. In the context of these children, it aids in understanding how societal perceptions influence their self-identity and overall well-being.*  *Mothers while recognized as the primary victims of genocide rape, find their children victimized as a consequence of the crimes perpetrated against them [15–17]. [18]These children, vulnerable to health and well-being hazards from conception, face heightened risks that may persist throughout their lives [18]. The study's imperative is to explore how the perceptions, beliefs and attitudes of the family and community toward these offspring, identified as the perpetrator's children, affects their mental health in adulthood, and hence reduced capacity to recover from mental health problems. Culturally, a child belongs to his father, not his mother [19], leading to societal biases and abuse [5,7], as Tutsi survivors label these children as Interahamwe. Meanwhile, [5,7][19]Hutu rapists' relatives frequently abuse the children's mothers for testifying against their fathers and sending them to jail.*  *Despite the majority of existing knowledge being derived from mothers' perspectives [7,20,22,23], this study aims to shift the focus towards understanding the experiences and coping strategies of these children from their viewpoints. By incorporating adaptive coping strategies[24], developed by scholars like Susan Folkman and Richard Lazarus, into our analysis, we strive to design effective interventions that prevent or mitigate the potential consequences of adverse life experiences related to being conceived through genocidal rape. This research also aims to contribute insights into breaking the cycle of intergenerational trauma by supporting the youth born of genocidal rape in their future parenting and parent-child relationships. Adaptive coping strategies refer to the conscious efforts individuals make to manage stressors effectively. In the context of children born of genocidal rape, understanding these adaptive coping strategies is crucial for designing interventions that can help them navigate the challenges stemming from their unique life experiences.*  *In a landscape where existing studies are often overly broad [21,22], this study specifically tailored to explore how the family and community attitudes and perceptions of the children conceived via genocidal rape and the offspring’s self-perception would affect the recovery from mental health problems. This study also sought to reveal how the offspring are coping with these perceptions and mental health problems. By comprehensively examining family and community attitudes, self-perception, coping mechanisms, and mental health recovery, this study seeks to pave the way for a more informed and targeted approach to support the individuals born of genocidal rape.* |
|  |  | 2. The paper's quality is elevated by the careful attention given to its methodology; however, including the age range of the participants in the Method section would add valuable detail and enhance study's applicability. |  |  |
|  |  | 3. According to my understanding, Line no. 200 ( Half of the mothers (8/16) is contradicting with the inclusion criteria described in Line 132 (were married). If others are widow then mention this also in inclusion criteria. |  | **Thanks for the comment. It was addressed as follow:**  The inclusion criteria were a single mother or widow of any age (i) who had a last-born offspring conceived from genocidal rape and ii) who has not been married since the rape |
|  |  | 4. Line 121-124 requires rephrasing as it is not giving a comprehensive understanding of the subject matter. please note some typing mistakes. Line 197: windowed should be widow Line 132: (i) should be (ii) |  |  |

| **N** | **Reviewer 2** | **Reviewer’s comments** | **Page and line on which addressed** | **How comments were addressed** |
| --- | --- | --- | --- | --- |
| 1 | **Title & Abstract** | Reviewer #2: The research titled "The Influence of Family and Community Perceptions on the Mental Health Prognosis of Offspring Born of Genocidal Rape" makes a noteworthy contribution to the existing body of knowledge pertaining to the mental health of individuals born of genocidal circumstances. Its distinctive perspective, methodological rigor, and actionable recommendations contribute to its significance. However, to enhance the study's effectiveness and applicability, certain areas for improvement have been identified. | Whole document | Thank you very much for the overall appreciation of the quality of this manuscript. |
|  |  | Primarily, the study's title implies a focus on offspring born of genocidal rape, yet the inclusion of mothers in the sample requires clarification of the rationale behind this decision. Explicitly addressing the purpose and relevance of involving mothers would contribute to the study's transparency and coherence. | Page 2, line 17 | **Thanks for the comment. It was addressed as follow:**  While the focus of this study was children, we felt that it was important to include moms to ensure response validity. When the children judged the intervention's usefulness and efficacy, we anticipated that moms would experience a spillover effect. |
| 2 | The introduction | There is also dearth of theoretical explanation of finding. By taking into account the theories may help to further explanation of the factors. |  | **We appreciate the reviewer for the constructive comments, we have included four different theories that may help to further explanation of the factors in our introduction as follows:**  ***Introduction***  *Rape was a major issue during the 1994 genocide against the Tutsi, and its results had long-term severe effects on the mental health and psychosocial living conditions of the victims and the offspring born of this sexual violence [1]. During the Rwandan genocide, men, predominantly Hutu, utilized the rape of women, primarily Tutsi, as a political weapon to exterminate the Tutsi ethnic group [2]. Drawing upon trauma theory[3], which illuminates the profound impact of traumatic events, we understand the enduring psychological wounds inflicted during this period. Originating from the works of psychoanalyst Sigmund Freud, trauma theory posits that exposure to severe, distressing events can have lasting psychological impacts on individuals. In the context of genocidal rape, trauma theory helps us understand the deep and lasting effects on the mental health of both the victims and their offspring, emphasizing the interconnectedness of individual and collective trauma.*  *Between April 6 and July 12, 1994, Tutsi girls and women endured widespread rape, particularly intensifying after mid-May when Hutu officials directed the Interahamwe ("those who stand together") militia not to spare Tutsi women and children in the genocide [2]. This period witnessed the utilization of genocidal rape to humiliate, kill and eradicate the Tutsi as an ethnic group [4,5]. Social cognitive theory, developed by Albert Bandura, provides a lens to analyse how family and community attitudes shape the self-perception of children born of genocidal rape, exploring the observational learning and socialization processes influencing their coping strategies. In the context of children born of genocidal rape, this theory helps us understand how their self-perception is influenced by the attitudes and behaviours they observe in their family and community.*  *Though the exact number of children born as a result of genocide rape remains unknown, it is estimated that between 2,000 and 10,000 offspring [6,7], were born from 250,000 to 500,000 estimated rapes of Tutsi women and girls [7,8]. Many of these children, born of multiple and gang rapes, find themselves disconnected from their fathers and the families from which they came [9]. The secrecy surrounding their origins, rooted in the mothers' difficulty in disclosing the circumstances of their children's birth, poses challenges [9]. Using ecological systems theory[10], developed by Urie Bronfenbrenner, we examine the multifaceted influences on mental health at individual, family, community, and societal levels. This theory posits that individuals are influenced by multiple interconnected systems, including the microsystem (individual relationships), mesosystem (interactions between microsystems), exosystem (external influences on microsystems), macrosystem (cultural values), and chronosystem (temporal changes). Applied to the context of children born of genocidal rape, this theory allows us to explore how various environmental factors at different levels impact their mental health and coping mechanisms.*  *[9]The offspring born of genocidal rape face profound challenges, including societal violent abuse resulting from their birth and the lack of stable familial networks [11]. Health issues and developmental challenges further compound their struggles, with mothers' psycho-social trauma influencing early childhood and adulthood development [11]. Stigmatization[12,13] and identity[14] theories, developed by Erving Goffman and Erik Erikson, respectively, shed light on how societal labels and perceptions impact the self-identity and well-being of these children. Stigmatization theory explores how individuals with socially stigmatized attributes are treated by society. Applied to the children born of genocidal rape, this theory helps us understand the societal biases and abuse they face due to being labelled as the perpetrator's offspring. Identity theory, on the other hand, emphasizes the stages of identity development individuals go through. In the context of these children, it aids in understanding how societal perceptions influence their self-identity and overall well-being.*  *Mothers while recognized as the primary victims of genocide rape, find their children victimized as a consequence of the crimes perpetrated against them [15–17]. [18]These children, vulnerable to health and well-being hazards from conception, face heightened risks that may persist throughout their lives [18]. The study's imperative is to explore how the perceptions, beliefs and attitudes of the family and community toward these offspring, identified as the perpetrator's children, affects their mental health in adulthood, and hence reduced capacity to recover from mental health problems. Culturally, a child belongs to his father, not his mother [19], leading to societal biases and abuse [5,7], as Tutsi survivors label these children as Interahamwe. Meanwhile, [5,7][19]Hutu rapists' relatives frequently abuse the children's mothers for testifying against their fathers and sending them to jail.*  *Despite the majority of existing knowledge being derived from mothers' perspectives [7,20,22,23], this study aims to shift the focus towards understanding the experiences and coping strategies of these children from their viewpoints. By incorporating adaptive coping strategies[24], developed by scholars like Susan Folkman and Richard Lazarus, into our analysis, we strive to design effective interventions that prevent or mitigate the potential consequences of adverse life experiences related to being conceived through genocidal rape. This research also aims to contribute insights into breaking the cycle of intergenerational trauma by supporting the youth born of genocidal rape in their future parenting and parent-child relationships. Adaptive coping strategies refer to the conscious efforts individuals make to manage stressors effectively. In the context of children born of genocidal rape, understanding these adaptive coping strategies is crucial for designing interventions that can help them navigate the challenges stemming from their unique life experiences.*  *In a landscape where existing studies are often overly broad [21,22], this study specifically tailored to explore how the family and community attitudes and perceptions of the children conceived via genocidal rape and the offspring’s self-perception would affect the recovery from mental health problems. This study also sought to reveal how the offspring are coping with these perceptions and mental health problems. By comprehensively examining family and community attitudes, self-perception, coping mechanisms, and mental health recovery, this study seeks to pave the way for a more informed and targeted approach to support the individuals born of genocidal rape.* |
| 3 | **Material and Methods** | While the research highlights the necessity for culturally relevant mental health interventions, it falls short in providing an in-depth exploration of the specific cultural nuances influencing the mental health of the studied population. A more thorough discussion of cultural factors is imperative to inform the development of interventions that resonate with the cultural context. Furthermore, the study lacks an exploration of external factors that could potentially contribute to the mental health outcomes of the offspring. Factors such as socioeconomic status, access to mental health resources, and ongoing socio-political conditions are integral components influencing mental health. A more comprehensive discussion of these external factors would fortify the study's contextual understanding and relevance. The research significantly contributes to the field, addressing these identified areas for improvement would strengthen its overall impact and align it more closely with best practices in academic research. |  | Thank you very much for the insightful comments. We agree with the reviewer that this study lacks an exploration of external factors that could potentially contribute to the mental health outcomes of the offspring. We found it imperative to include that in the study limitations (third one):  ***Study limitations***  *This study has numerous limitations that should caution against generalizing the findings. First, as with any self-report data, information acquired from the participants was impacted by their willingness to share personal information. Because of probable shame and blame, some of the respondents may have been unable to communicate their experiences. Second, the sample may be overrepresented the mothers and their offspring with disadvantaged Rwandan women with poor socioeconomic status as they were beneficiaries of SURF Rwanda. Therefore, there are no viewpoints from individuals from a high socioeconomic class.* ***Third, the study lacks an exploration of external factors such as socioeconomic status, access to mental health resources, and ongoing socio-political conditions that could potentially contribute to the mental health outcomes of the offspring. Future studies exploring these potential factors are warranted****. Finally, the qualitative methodology of the study restricts the generalizability of the findings.* |
